# Expanding the Analytical Toolbox for Extracellular Vesicle Biochemical Profiling: A Multiplatform Spectroscopic and Chromatographic Strategy

**DOI:** 10.1021/acs.analchem.5c07632

**Published:** 2026-04-24

**Authors:** Caterina Branca, Angela Paterna, Estella Rao, Samuele Raccosta, Mohamed Zekri, Sabrina Picciotto, Paola Gargano, Giorgia Adamo, Luana Pulvirenti, Laura Siracusa, Antonella Bongiovanni, Mauro Manno

**Affiliations:** † Department of Mathematical and Computational Sciences, Physical Science and Earth Science, 18980 University of Messina, 98166 Messina, Italy; ‡ 567333 Institute of Biophysics, National Research Council of Italy, 90146 Palermo, Italy; ¶ Institute for Biomedical Research and Innovation, National Research Council of Italy, 90146 Palermo, Italy; § Institute of Biomolecular Chemistry, National Research Council of Italy, 95126 Catania, Italy

## Abstract

The molecular characterization of extracellular vesicles (EVs) remains a major analytical challenge, particularly regarding lipid components that govern vesicle stability, biogenesis, and biological activity. Here we present an integrated workflow combining high-performance liquid chromatography with diode array detection (HPLC-DAD), attenuated total reflection Fourier transform infrared spectroscopy (ATR-FTIR), and gas chromatography–mass spectrometry (GC–MS) to profile EV lipids from both mammalian (human embryonic kidney HEK293T human cell line) and microalgal (*Tetraselmis chuii*) cell-derived EVs. Quantitative phospholipid analysis by HPLC-DAD and fatty acid profiling by GC–MS revealed phosphatidylserine as a conserved and selectively enriched lipid signature in EVs across phylogenetically distant species, along with system-specific differences in acyl chain saturation. FTIR spectroscopy was extended beyond conventional biochemical fingerprinting to derive spectroscopic indicators (including protein-to-lipid ratio, saturated/unsaturated balance, acyl chain length, and lateral packing) that are effectively related to EV sources. This multitechnique approach provides comprehensive structural readouts that translate complex molecular diversity into reproducible descriptors of extracellular vesicle identity and function. Our findings also identify distinctive lipid and spectroscopic signatures in microalgal EVs, reinforcing their potential as sustainable nanobiotechnological platforms.

## Graphical abstract

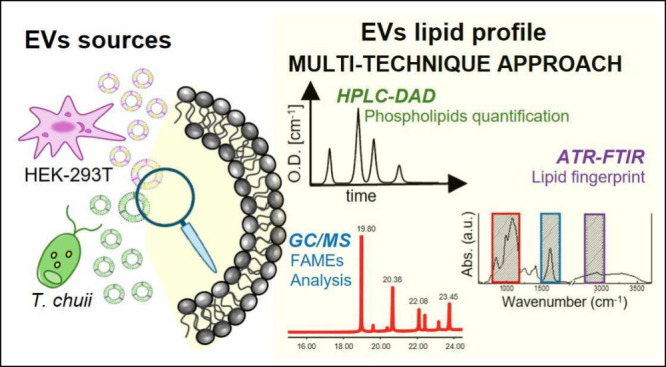

## Introduction

Extracellular vesicles (EV) are nanostructures enclosed in lipid bilayers secreted by virtually all living cells, mediating intercellular communication through the transfer of proteins, nucleic acids, and metabolites.^1^ Their identity and role in physiological and pathological pathways are determined by their physicochemical properties, such as size, electric charge, stiffness, as well as by their biochemical composition, including the protein and nucleic acid content.^2,3^ In this context, the lipid analysis is relevant to provide further insight into the origin of the vesicles in EVs-enriched samples. Also, the lipid composition remains critical, as lipids not only ensure structural integrity but also contribute to bilayer asymmetry, membrane curvature, and domain organization^4^ The EV membrane is not a simple replica of the parental cell’s lipid bilayer but is characterized by a unique structure^5^ This distinctive composition involves the selective enrichment of certain lipid classes, such as phosphatidylserine, sphingomyelin, and cholesterol, supporting the hypothesis that lipid sorting during vesicle biogenesis is an active and regulated process.^5–7^ These features strongly influence vesicle stability, uptake, and function.

The most recent MISEV guidelines^2,8^ highlight the importance of lipid characterization and report several approaches for total lipid quantification, including fluorescent intercalating dyes, attenuated total reflection Fourier-transform infrared (ATR-FTIR) spectroscopy, and the sulfo-phospho-vanillin assay.^9^ Yet, these methods remain limited in their sensitivity or selectivity, raising concerns about their ability to fully capture the complexity of EV lipidomes. Mass spectrometry (MS) is widely regarded as the gold standard for detailed lipidomics,^9,10^ providing unparalleled resolution and coverage. Nevertheless, MS requires expensive instrumentation, specialized expertise, and extensive sample preparation, which may hinder its adoption in routine laboratory workflows and in large-scale production and quality control.

To address these challenges, complementary methods are needed that can provide rapid, reproducible, and accessible insights into EV composition. Indeed, genomics, proteomics, glycomics, and lipidomics provide detailed quantitative information on EV molecular profiling but involve complex sampling procedures that can alter the ratio among different molecular classes. In contrast, ATR-FTIR spectroscopy, in particular, holds promise due to its ability to provide label-free, nondestructive, and semiquantitative information on the relative contributions of lipids, proteins, nucleic acids, and other biomolecules in intact vesicle preparations,^11–13^ as well as in cells or complex biological systems.^14,15^ Although FTIR has occasionally been applied to EVs, its use has been largely confined to general biochemical fingerprinting, with limited exploration of lipid-specific features.^16^ Recent studies have nevertheless demonstrated the capability of ATR-FTIR to detect compositional differences among EV subpopulations and disease-associated molecular alterations, including changes in protein secondary structure, lipid organization, and glycosylation patterns.^13,17^

Here, we extend the application of FTIR in a more systematic and EV-focused manner, complementing it with phospholipid quantification by high performance liquid chromatography with diode array detection (HPLC-DAD) and fatty acid profiling by gas chromatography mass spectrometry (GC-MS).Rather than proposing FTIR as a stand-alone replacement for lipidomics, the present study addresses the hypothesis that FTIR-derived structural descriptors can capture biologically meaningful differences in EV membrane organization and complement chromatographic and MS-based analyses.

In this study, we applied this combined approach to EVs from the microalgae *Tetraselmis chuii* (*T. chuii*) and EVs derived from mammalian HEK293T cells.This comparison was designed to assess whether conserved lipid-sorting features coexist with source-dependent membrane structural signatures that can be resolved through integrated spectroscopic and chromatographic analyses.Although EVs have been extensively characterized in mammalian systems for their physicochemical properties, biochemical profile, and roles in physiological and pathological processes, EVs from microalgae, called nanoalgosomes, have been identified and studied quite recently.^18–20^ Nanoalgosomes are emerging as promising biogenic nanocarriers due to their biocompatibility, cost and environmental sustainability, and potential applications in biotechnology and drug delivery.^21–24^

Our results reveal significant differences between vesicles and their parental cells, with phosphatidylserine emerging as a conserved and selectively enriched lipid in both biological systems. These findings reinforce the concept of lipid sorting as a tightly regulated aspect of vesicle biogenesis and identify molecular features potentially shared across phylogenetically distant EV populations. Moreover, the identification of lipid signatures specific to nanoalgosomes offers new insights into their physiological roles and underlines their potential in sustainable nanobiotechnologies.

By performing an accurate analysis of ATR-FTIR spectra, some relevant spectroscopic indicators emerge: (1) the Protein to Lipid ratio (P/L), that elicit the lipid amount with respect to the protein amount, easily accessible by chromatographic assays; (2) the Saturated Fatty Acids to Unsaturated Fatty acid ratio (SFA/UFA), that points out the quality and potential function of EVs; (3) the Acyl chain length (ACL), an index that refers to number of carbon atoms in the tail of fatty acids, a parameter affecting the bilayer physical properties, including membrane thickness and plasticity; (4) the orthorhombic to hexagonal phase ratio (OR/HEX), a parameter that indicates the degree of order in the lipid bilyer.

This multitechnique framework is intended to broaden the analytical toolbox available for EV research, highlighting the complementary value of approaches that are cost-effective, scalable, and well suited for comparative studies across diverse EV sources.

## Materials and Methods

### Chemicals

The reagents used for HPLC analysis were HPLC-grade. Acetonitrile, Methanol, and 2-Propanol were purchased from Sigma-Aldrich Chemical Co., phosphoric acid solution 49–51% was purchased from Fluka Analytical. Deionized water was used. Phospholipid mixture for HPLC from *Glycine max* (soybean) in chloroform were supplied by Sigma-Aldrich Chemical: 2 mL, containing L-α-Lysophosphatidylcholine 3 mg mL^–1^ (LysoPC), L-α-Phosphatidylcholine 1.5 mg mL^–1^ (PC), L-α-Phosphatidyl­ethanolamine 1.2 mg mL^–1^ (PE), L-α-Phosphatidylinositol sodium salt 9 mg mL^–1^ (PI)), L-α-Phosphatidyl-serine (PS) and sphingomyelin (SM). The following analytical standards (all purchased from Sigma-Aldrich (Merck Italy, Milan) were used for peak identification in GC-MS experiments: Methyl palmitoleate, Methyl palmitate, Methyl cis,cis-9,12-octadecadienoate, Mehtyl oleate, Methyl stearate, all-cis- 4,7,10,13,16,19-docosahexaenoic acid methyl ester.

### Microalgal-Derived EVs Isolation from *T. chuii* Culture

The marine green algae *Tetraselmis chuii* (*T. chuii*) (CCAP 66/21b) was cultured in F/2 medium with 20 nm filtered seawater, under sterile conditions, continuous aeration, and a 14:10 light-dark cycle at 19 ± 1 °C for 4 weeks. Growth was monitored using optical density measurements as reported in Paterna et al., 2022. Each batch of nanoalgosomes has been isolated from *T. chuii* cultures using tangential flow filtration following the protocol outlined in Paterna et al., 2022.

### EV Isolation from HEK293T Cell Culture

#### Cell Culture

Human embryonic kidney (HEK) 293T cells were cultured in high-glucose Dulbecco’s Modified Eagle Medium (DMEM, Sigma-Merck, Cat. No. D6429) supplemented with 10% fetal bovine serum (FBS), 1% penicillin-streptomycin and 1% l-glutamine. Cells were kept at 37 °C in a humidified atmosphere containing 5% CO_2_. When cultures reached approximately 50% confluency, the growth medium was replaced with fresh DMEM containing 10% EV-depleted FBS, 1% penicillin–streptomycin and 1% l-glutamine. After 48 h of incubation, 50 mL of conditioned medium were collected. EV isolation was achieved through an integrated approach that combined differential centrifugation (dC), tangential flow filtration (TFF), and size-exclusion chromatography (SEC).

#### dC

Differential centrifugation was first employed to efficiently remove cells, cellular debris, and larger particles. The medium was subjected to sequential centrifugation steps: 300 × *g* for 10 min at 4 °C (twice), followed by 2000 × *g* for 10 min at 4 °C (twice), and subsequently 10000 × *g* for 30 min at 4 °C.

#### TFF

Subsequently, the resulting supernatant was concentrated by TFF with a 500 kDa molecular weight cutoff hollow fiber filter (C02-E500–05-N, Repligen) to a final volume of 5 mL. TFF was used to concentrate the EV-containing supernatant and eliminate low-molecular-weight contaminants while preserving vesicle integrity.

#### SEC

Finally, high-performance size exclusion chromatography was performed to achieve high-purity isolation by removing residual protein contaminants and enabling buffer exchange. The procedure employed a manually packed XK 16/20 column (inner diameter: 16 mm, length: 20 cm; GE Healthcare Life Sciences) filled with Sepharose CL-2B resin (bed height: 18 cm), and operated with a Vanquish UHPLC system equipped with quaternary pump (VC-P20-A-01), split loop autosampler (VC-A13-A-02), UV–vis photodiode array detector (VC-D11-A-01), integral fraction collector (VF-F20-A-01). An isocratic separation was performed using Dulbecco’s PBS as the mobile phase, at a constant flow rate of 1 mL min^–1^. A 1 mL sample was injected onto the column,and different fractions of the elution volume were collected, each with a 1 mL volume; the fraction corresponding to EVs was detected by UV absorption at 254 nm.

### EVs Characterization

#### Dynamic Light Scattering (DLS)

A defined volume of the vesicle suspension was centrifuged at 1000 × *g* for 10 min at 4 °C to remove potential particulate contaminants. The resulting supernatant was carefully transferred, using Milli-Q-rinsed pipet tips, to a clean quartz cuvette and equilibrated at 20 °C within the thermostated chamber of a BI200-SM goniometer (Brookhaven Instruments). The instrument was configured with a He–Ne laser source (λ = 633 nm, JDS Uniphase 1136P) and a single-photon counting detector (Hamamatsu C11202–050). Dynamic light scattering (DLS) measurements were carried out by acquiring the intensity autocorrelation function *g*_2_(*t*) at a fixed scattering angle, ϑ = 90°, using a digital BI-9000 correlator (Brookhaven Instruments). The scattered intensity and its temporal correlation provide insight into the vesicle size distribution *P*_*q*_(σ) through the relation:

1 $$g_{2}\left(t\right)=1+{\left|\beta \int P_{q}\left(\sigma \right)e^{-D\left(\sigma \right)q^{2}t}d\sigma \right|}^{2}$$

where β is the coherence factor of the detection system, *q* = (4*πn*/λ)­sin­(ϑ/2) represents the magnitude of the scattering vector, with n = 1.3367 being the refractive index of the solvent. The diffusion coefficient *D*(σ) corresponding to vesicles of hydrodynamic diameter σ is calculated using the Stokes–Einstein relation:

2 $$D\left(\sigma \right)=\frac{k_{B}T}{3\pi \eta \sigma }$$

in which *k*_B_ is Boltzmann’s constant, *T* is the absolute temperature and η the dynamic viscosity of the dispersion medium. The function *P*_*q*_(σ) was reconstructed under the assumption that the underlying distribution of the diffusion coefficient *P*_*q*_(*D*) is the Schultz distribution, an asymmetric model of two parameters defined by a mean diffusion coefficient *D*_*z*_ and a variance *V*:

3 $$P_{q}\left(D\right)=\frac{\alpha }{D_{z}\Gamma \left(\alpha \right)}{\left[\frac{\alpha D}{D_{z}}\right]}^{\alpha -1}\exp\left(-\frac{\alpha D}{D_{z}}\right)$$

where α^–1^ is the normalized variance α^–1^ = *VD*_*z*_^–2^ This approach offers robustness against experimental noise and enables the extraction of key physical descriptors, namely: σ_*z*_ = *k*_B_ *T*/(3πη*D*_*z*_), the z-average hydrodynamic diameter, PDI, the polydispersity index, calculated as *PDI* = α^–1^ which quantifies the breadth of the size distribution.

#### Nanoparticle Tracking Analysis (NTA)

The concentration and size distribution of the nanoparticles were evaluated using a NanoSight NS300 (Malvern Panalytical, UK). EVs were diluted in HPLC-grade water (Sigma-Aldrich) previously filtered by 20 nm filters (Whatman Anotop) to obtain 20–120 particles per frame. Five 60-s videos per sample were recorded in light scattering mode at a syringe speed of 60, with camera settings adjusted to level 15–16. The recorded data were processed using NanoSight Software NTA 3.1 Build 3.1.46, applying the appropriate detection threshold.

#### Atomic Force Microscopy (AFM)

A 30 μL EV solutions with a concentration of 10^11^ particles mL^–1^, were deposited on glass slides functionalized according to the protocol used in Rao et al. 2025.^24^ Upon overnight incubation at 4 °C samples were gently rinsed with PBS. AFM measurements were carried out in PBS and in Quantitative Imaging mode by using a Nanowizard III scanning probe microscope (JPK Instruments AG, Germany) equipped with a 15 μm z-range scanner and AC40 (Bruker) silicon cantilevers (spring constant 0.1 N m^–1^, typical tip radius 8 nm). The 2 × 2 μm^2^ images were acquired at a force set point of 110 pN (z-length: 50 and 80 nm, pixel time: 5 and 8 ms for nanoalgosomes and HEK derived vesicles, respectively) and at a pixel size of 10 nm. The cantilever was thermally calibrated by using the tool in JPK software (version 4.2)^25^

#### BCA Assay

The Pierce BCA Protein Assay Kit (Thermo Fisher Scientific) was used to determine the protein concentration of the EVs. The absorbance of the BCA protein complex was recorded at 560 nm, according to the manufacturer’s instructions, using the Bio-Rad iMark microplate absorbance reader. The concentration is calculated by comparing the sample absorbance with that of a protein standard (bovine serum albumin, BSA, purchased from Sigma-Alsrich). A calibration curve was properly prepared by dilution of BSA.

#### Immunoblot Analyses

Protein separation of nanoalgosome, HEK-EVs proteins and HEK293-T cells lysate was performed using sodium dodecyl sulfate polyacrylamide gel electrophoresis (SDS- PAGE) (10%). 2× SDS sample loading buffer (0.2–0.1 M Tris-Cl pH 6.5, 4–8% (w/v) SDS, 20–40% glycerol 4.3 M, 2–4% bromophenol blue, 10–20% 2- mercaptoethanol) was added to 15 μg of EVs (in terms of total EV protein content, as determined with BCA) in Dulbecco’s PBS or 20 μg of lysate in RIPA lysis buffer. The samples were denatured at 100 °C for 7 min, loaded onto SDS–PAGE, and run at 100 V. Proteins were transferred onto methanol-activated PVDF membranes equilibrated in transfer buffer. The membrane was incubated overnight at 4 °C with the antibody anti H^+^-ATPase (diluted 1:1000 in blocking buffer, diluted 1:1 with TBS/Tween 0.2%, Agrisera, AS07260), antibody anti Alix (diluted 1:200 in blocking buffer, diluted 1:1 with TBS/Tween 0.2%, Santa Cruz Biotechnology Inc., sc-53538), CD63 Polyclonal Antibody (diluted 1:500 in blocking buffer, diluted 1:1 with TBS/Tween 0.2%, Invitrogen, PA5-92370) and antibody anti Calnexin (diluted 1:500 in blocking buffer, diluted 1:1 with TBS/Tween 0.2%, Santa Cruz Biotechnology Inc., sc-23954). The membrane was washed and incubated with goat anti-rabbit or mouse secondary antibody (dil 1:5000 in blocking buffer, diluted 1:1 with TBS/Tween 0.1%, LICOR IRDye 680RD or 800 RD) for 1 h at room temperature. Protein bands were detected using the Odyssey DLx Imager and its associated software (LI-COR, USA).

### Lipid Extraction and Analysis

#### HEK293T- and Microalgal-Derived EVs and HEK293T Cells Lipid Extraction Protocol

All samples analyzed were lyophilized using a freeze-dryer (ALPHA 1-2 LDplus, Martin Christ, Germany) prior to lipid extraction. Lipid extraction from the lyophilized EVs and cells was performed following the method of Bligh and Dyer.^26^ Freeze-dried preparations were suspended in a solvent mixture of chloroform:methanol:water (1:2:0.8, v/v/v), followed by sequential extractions with chloroform:water (1:1, v/v) and two additional extractions with pure chloroform. After each extraction step, samples were vortexed and centrifuged at 2000 × *g* for 10 min. The organic phases were collected, combined, and dried over anhydrous sodium sulfate (*Na*_2_ *SO*_4_), and the solvent was removed under a stream of nitrogen. A total lipid extract of approximately 2 mg was obtained. Each extract was subsequently dissolved in the appropriate volume of *n*-hexane:2-propanol (3:1, v/v) for further analysis.

#### Microalgae Lipid Extraction and Chlorophyll Removal

Microalgal culture (50 mL) was harvested by centrifugation at 1000 × *g* for 10 min. The resulting pellet was washed by resuspension in deionized water followed by a second centrifugation step under the same conditions. The wet biomass was then frozen at −80 °C and subsequently lyophilized. Approximately 60 mg of freeze-dried biomass were extracted using a biphasic solvent system composed of CHCl_3_:MeOH:H_2_O (1:2:0.8, v/v/v), followed by sequential extractions with CHCl_3_:H_2_O (1:1, v/v) and two additional extractions with pure CHCl_3_. After each extraction, samples were vortexed and centrifuged at 2000 × *g* for 10 min. The organic phases were pooled, dried over anhydrous sodium sulfate (*Na*_2_ *SO*_4_), and the solvent removed under a gentle stream of nitrogen. The crude lipid extract (8 mg) was subjected to solid-phase extraction (SPE) on a polyamide column to remove chlorophylls. The column was preconditioned with 5 mL of deionized water (Fraction 0) and eluted with a stepwise gradient of methanol in water as follows: 5 mL each of 1:8 MeOH:H_2_O (Fraction 1), 1:4 MeOH:H_2_O (Fraction 2), 2:3 MeOH:H_2_O (Fraction 3), 4:1 MeOH:H_2_O (Fraction 4), and 100% *MeOH* (Fraction 5). The pooled Fractions 1–4 were evaporated to dryness and kept at −20 °C until use.

#### UHPLC-UV-DAD Analysis and Quantitative Determination of Phospholipids

Vanquish UHPLC System, Thermo Scientific Ultra High-Performance Liquid Chromatography (UHPLC) has been performed in order to separate phospholipids using a modular Thermo Vanquish UHPLC system equipped with a quaternary pump (VC-P20-A-01), a split loop autosampler (VC-A13-A-02) and an UV–vis photodiode array detector (VC-D11-A-01). The column compartment (VC-C10-A-03) was used to control the column temperature. Data acquisition and analysis were carried out using Chromeleon software (Thermo Scientific). Further analysis was carried out using the General Public License software XMGRACE (https://plasma-gate.weizmann.ac.il/Grace). The phospholipid separation protocol was adapted to a previously reported protocol (Rehman et al., 2017). A normal phase analytical column Kromasil SILICA 5 μm 100Å (4.6 mm × 250 mm) was used. The isocratic separation of the phospholipids was achieved with a mobile phase of ACN:MeOH:phosphoric acid (100:10:1.8, v/v/v). Twenty μL of samples was injected applying a flow rate of 0.5 mL min^–1^, with the pressure of ca. 19 bar. Measurements were performed at 25 °C, to ensure repeatability; the detector wavelength was set at 203 nm. For a quantitative phospholipid determination the following pure lipid standards were were dissolved in *n*-Hexane:2-Propanol (3:1, v/v) and injected in the columns at different concentrations: PE (15–150 μg mL^–1^), PC (20–200 μg mL^–1^), LysoPC (3.5–35 μg mL^–1^), PS (34–340 μg mL^–1^), SM (17–170 μg mL^–1^), LysoPE (34–340 μg mL^–1^).

#### ATR-FTIR Spectroscopy

ATR-FTIR spectra were recorded at room temperature in the mid-IR range (400–4000 cm^–1^) on a Bruker Vertex 80 V FTIR spectrometer equipped with a Bruker Platinum ATR accessory with a single reflection diamond crystal. Each spectrum was averaged over 128 scans with a resolution of 4 cm^–1^. The sample solution droplets were deposited on the ATR crystal and a thin film was obtained by slow evaporation of the solvent under ambient conditions. A background scan was recorded prior to the measurement and subtracted from the sample spectra. The measurements were repeated for each sample to assess reproducibility. The spectra were registered and preprocessed using commercial OPUS software. Spectra were normalized after linear baseline subtraction. To resolve overlapping features within specific spectral regions of interest, we first applied the second derivative of the spectra using OriginPro2019 software with a 7-point Savitzky–Golay smoothing with polynomial order of 2. This is generally the most appropriate choice for FTIR measurements recorded with a resolution of 4 cm^–1^ since this window size minimizes spectral distortion, allowing for a more accurate discrimination of the overlapped bands and identification of the local minima. However, in spectral regions with particularly high complexity, the fourth derivative proved especially advantageous. By enhancing subtle inflection points, this method facilitates the detection of weak or hidden peaks that may not be discernible using lower-order derivatives. In parallel, we applied iterative peak-fitting deconvolution of the absorbance spectra using the PeakFit software package. The spectra were decomposed into a set of Gaussian components, with the fit optimized by minimizing both the χ^2^ statistic and the residual sum of squares (RSS). No constraints were imposed on the fitting parameters for this analysis. Interestingly, regardless of the approach used (either derivative analysis or curve fitting deconvolution), the results revealed the same number of distinct peaks for each spectral band investigated. Moreover, a consistent match was observed in the central frequencies, within the limits of experimental error. The good agreement between the two independent approaches confirms the reliability of the results achieved. As a final remark, we want to highlight the clear intragroup spectral similarity observed in the full FTIR-ATR spectra of both Algal and HEK-EVs. For this reason, in the next section we will present for different regions only one representative spectrum per species.

#### Fatty Acids Derivatization and GC-MS Analysis

EV lipid extracts, prepared as described above, from 3 nM of lyophilized EVs stored at −80 °C, were allowed to reach room temperature and directly subjected to mild methylation to obtain fatty acid methyl esters (FAMEs). The derivatization method, adapted from official protocols^27^ and optimized in-house for this biological matrix, was selected to minimize degradation of thermolabile or chemically sensitive components. Approximately 10 mg of thawed EVs extract were weighed into 8 mL amber vials fitted with screw caps and silicone/PTFE septa (Agilent Technologies, Milan, Italy). A volume of 200 μL *n*-hexane and 20 μL of 2 N KOH in methanol (prepared by dissolving 11.2 g KOH in 100 mL methanol) was added. The biphasic mixture was stirred magnetically for 2 min at room temperature, then left to stand for at least 2 h to ensure full phase separation. The upper hexane layer was carefully collected using a glass Pasteur pipet, dried over anhydrous sodium sulfate, filtered through Whatman No. 1 paper, and transferred to 2 mL GC autosampler vials for subsequent analysis. All methylation steps were performed in duplicate.

Gas chromatographic analyses of FAMEs were performed in fast mode using a PerkinElmer Clarus 690 FID system with a flame ionization detector (FID) and TcNAV Chromatography Data System v6.3.4 software. The system was equipped with a PerkinElmer ELITE-5MS capillary column (30 m × 0.25 mm × 0.25 μm), Hydrogen as carrier gas, injection in split mode (1:20), 5 μL injection volume, injector at 250 °C, detector at 280 °C, and linear carrier gas velocity set at 20 mL min^–1^. Oven temperature was initially held at 60 °C for 4 min, followed by a programmed ramp to 280 °C at 10 °C min^–1^ for a total run time of 5 min. Compound quantification was based on GC-FID peak area percentages. For mass spectral identification, analyses were repeated using a PerkinElmer Clarus SQ 8 T instrument under fast GC–MS conditions with hydrogen as the carrier gas and a PerkinElmer ELITE-5MS capillary column (30 m × 0.25 mm × 0.25 μm). The injector and detector temperatures were 250 and 280 °C respectively, with split mode injection (1:50), 5 μL volume, and column flow at 20 mL/min. Ionization was performed by electron impact (70 eV), with the electron multiplier set to 1000 V and transfer line maintained at 280 °C. Compound identification was based on comparison of mass spectra with those in the NIST 20 library, as well as published literature. All analyses were carried out in triplicate on three independent biological samples.

## Results and Discussion

### EV Characterization

EVs were isolated from microalgae *T. chuii* and from HEK293T, a mammalian cell line.As evidenced in the Methods section, the isolation protocols are designed to select what are commonly referred as small EVs, both for TFF isolation, where a 200 nm cutoff is used, and for the dUC isolation, where large objects are pelleted (and discarded) by a 10 kg centrifugation.Quality control checks have been conducted on at least three batches of EV preparations (different biological replicates) to ensure the reproducibility of theisolation process.Routinary biochemical and biophysical analyses have been performed as outlined in our previous work in accordance with the MISEV guidelines.^2,18^ The size distribution was measured by dynamic light scattering (DLS) and nanoparticle tracking analysis (NTA). In particular, DLS can determine the entire size distribution in a sample of nanoparticles from a few nanometers to hundreds of nanometers, thus eliciting whether the sample also contains small molecules or large aggregates along with the expected population of EVs.^18^ We analyze the intensity autocorrelation functions (inset of Figure 1A,E) using the Shultz distribution for the diffusion coefficient that is, a two-parameter distribution, whose skewness and, in general, all high-order cumulants are determined uniquely by the average σ and the variance. This minimalistic approach, admitted by the typical noise level and in the experimental autocorrelation functions, is a key point for deriving useful and readable information from DLS experiments, better than the classical regularization methods.^28^ In comparison, NTA is less definitive in determining the actual size distribution (Figure 1B,F), as it is more accurate in measuring large particles.^18^ Nevertheless, NTA remains very useful to obtain a direct estimate of particle concentration. Another method of determining the amount of nanoparticles is to measure their total protein content; we used the typical BCA colorimetric assays. The EV particle number/protein ratio that is 1 μg of total EV protein corresponds toan order of magnitude of10^10^ particles in all EV batches, is coherent with the estimate of EV particles/μg of EV proteins, as reported by Sverdlov (2012)^29^ and assessed for nanoalgosomes in our previous work.^21^ In particular, in the bathes used in the present work, we obtained the following particle concentrations (by NTA) and protein concentrations (by BCA): for microalgal EVs 1500 ± 130, 2700 ± 100, 1900 ± 60 × 10^–9^ particles/mL and 130 ± 2, 80 ± 3, 62 ± 3 μg/mL, respectively; for HEK-EVs 410 ± 30, 36 ± 2 × 10^–9^ particles/mL and 9.3 ± 0.3, 4.4 ± 0.3 μg/mL, respectively.

**Figure 1:**
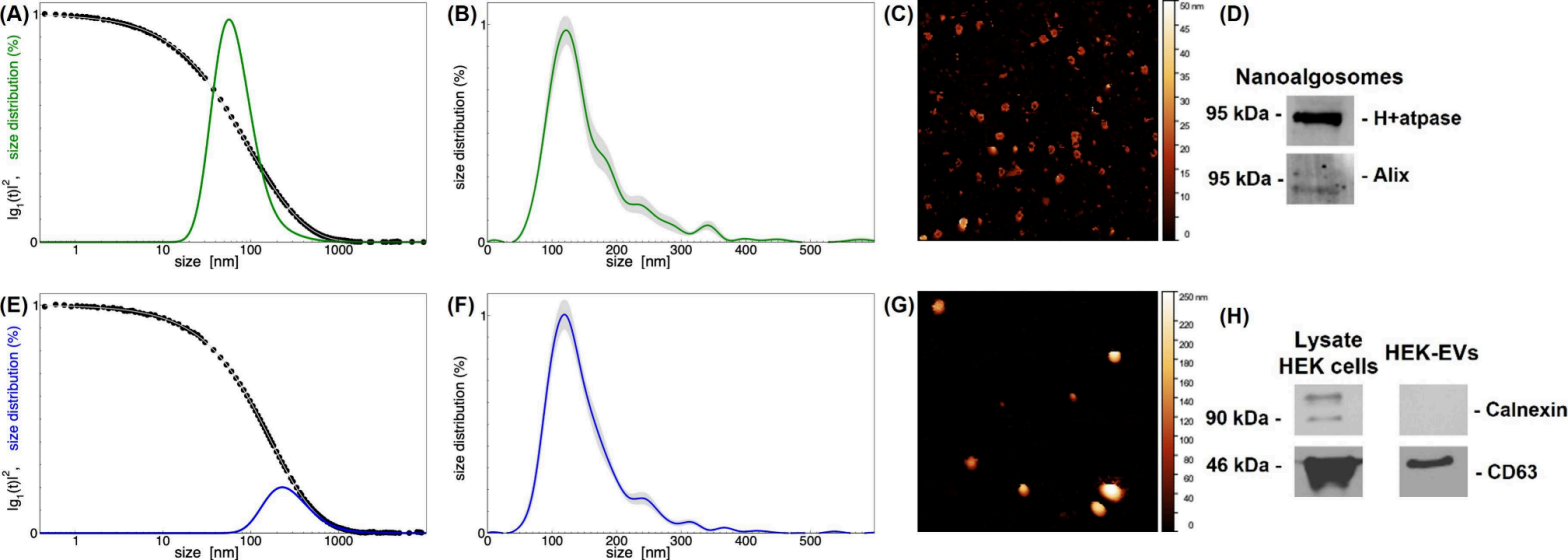
Extracellular vesicle characterization: (A–D) nanoalgosomes; (E–H) HEK-EVs. (A, E) DLS: intensity autocorrelation functions (circles), data fitting (dashed curves), size distributions (bold solid curves). (B, F) NTA: size distributions. (C, G) AFM: 2 × 2 μm images; (D, H) immunoblot analyses.

Atomic force microscopy images were taken to assess EV morphology and integrity, and at the same time check the purity of sample preparations (Figure 1C,G). The AFM images confirm that the EV samples are clean and homogeneous, with a negligible presence of smaller objects.

Immunoblot analyses were performed on selected biomarkers for the two EV types. In particular, nanoalgosomes were analyzed to detect the EV-associated markers H^+^-ATPase and Alix [Figure 1(D)] and HEK-EVs were analyzed to assess the presence of CD63, and at the same time the absence of calnexin, used as a negative control, in comparison to the HEK293T cell lysate [Figure 1(H)]

### Phospholipid Profile by HPLC-DAD

#### Calibration Curves

To enable quantitative analysis of the phospholipid composition in lipid extracts obtained from cells or EVs samples, mixtures of phospholipid standards were analyzed under identical chromatographic conditions. The identification of phospholipids was carried out by comparison with the retention time of pure standards, all injected at the same chromatographic conditions (Figure 2A). The corresponding retention times for the target phospholipids are reported in Table 1.

**Figure 2:**
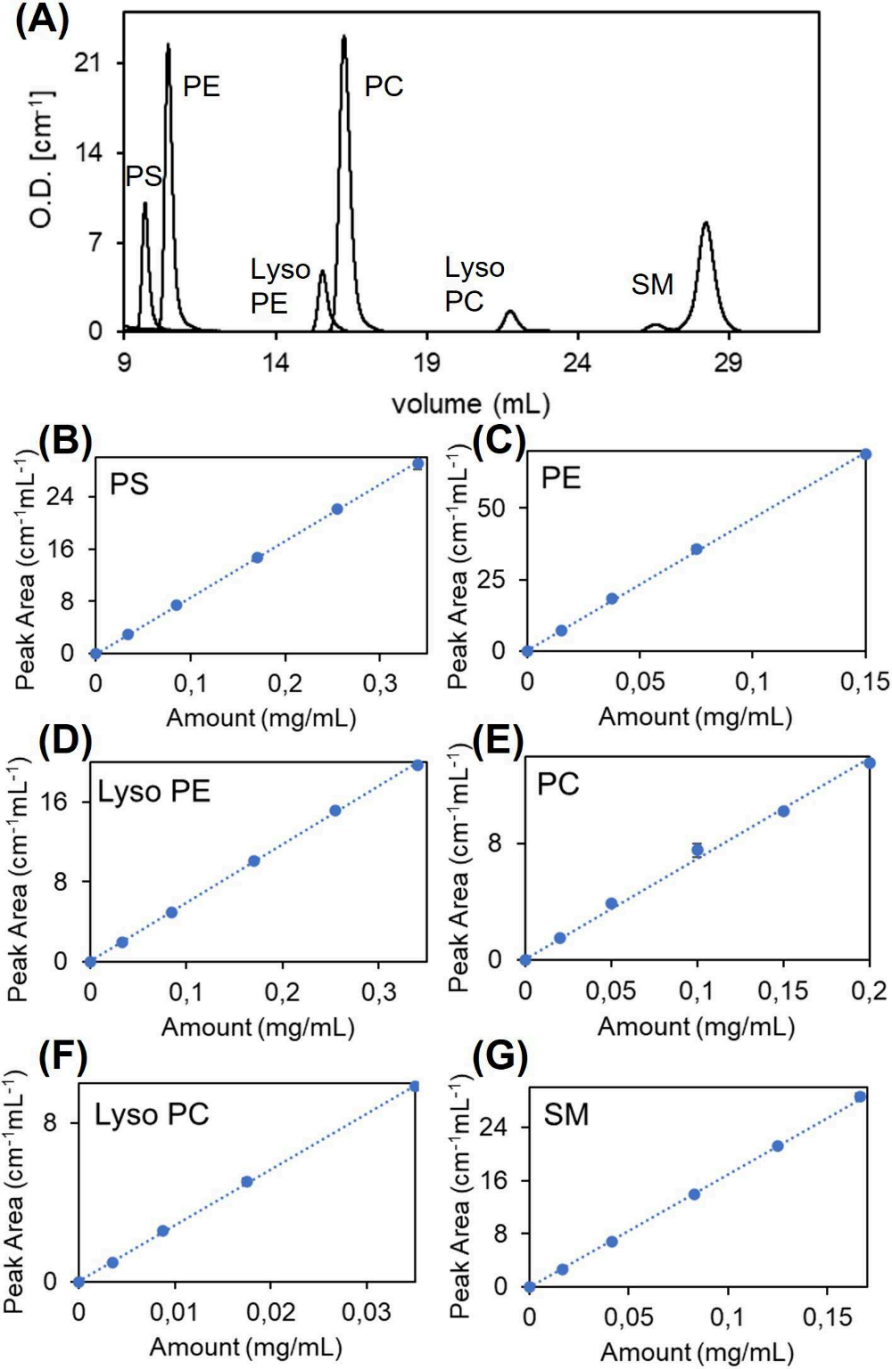
Lipid standards calibration for HPLC-DAD experiments. (A) Representative chromatogram showing the separation of the lipid standards used for quantification (UV absorbance at 203 nm). (B–G) Calibration curves for lipid classes (PC, PE, PS, LysoPC, LysoPE, and SM), based on serial dilutions of known concentrations.

**Table 1:** Lipid Standards Calibration Parameters

Phospholipid standard	Retention volume (mL)	Area coefficient (μg mL^–1^)
PS	9.8 ± 0.3	11.58 ± 0.07
PE	10.5 ± 0.5	2.16 ± 0.02
LysoPE	15.5 ± 0.3	17.07 ± 0.14
PC	16.0 ± 0.3	1.90 ± 0.02
LysoPC	22.5 ± 0.3	3.54 ± 0.03
SM	28.0 ± 0.4	5.88 ± 0.03

For each phospholipid standard, decreasing volumes (20–2 μL) of concentrated standard solutions were injected in triplicate into the column and the area of the absorption peak at 203 nm was calculated. The calibration curves, generated by plotting the peak area as a function of the concentration, exhibit a high linearity within the defined concentration ranges (Figure 2B–G), thus enabling accurate quantification of the phospholipid species in the cells or EV-derived lipid extracts. The calibration coefficients were derived by data linear regression (Figure 2B–G).

#### Lipid Extraction

The extraction of the lipid component was achieved by a slightly modified version of the classical Bligh and Dyer protocol. As a first step, the samples were carefully lyophilized to minimize the loss of vesicles and increase the lipid extraction efficiency; indeed an accurate removal of water content enable a more efficient dilution into the organic solvents. The lipid component was then collected by liquid–liquid partitioning, after several washing steps, as described in the Materials and Methods section and shown in Figure 3A.

**Figure 3:**
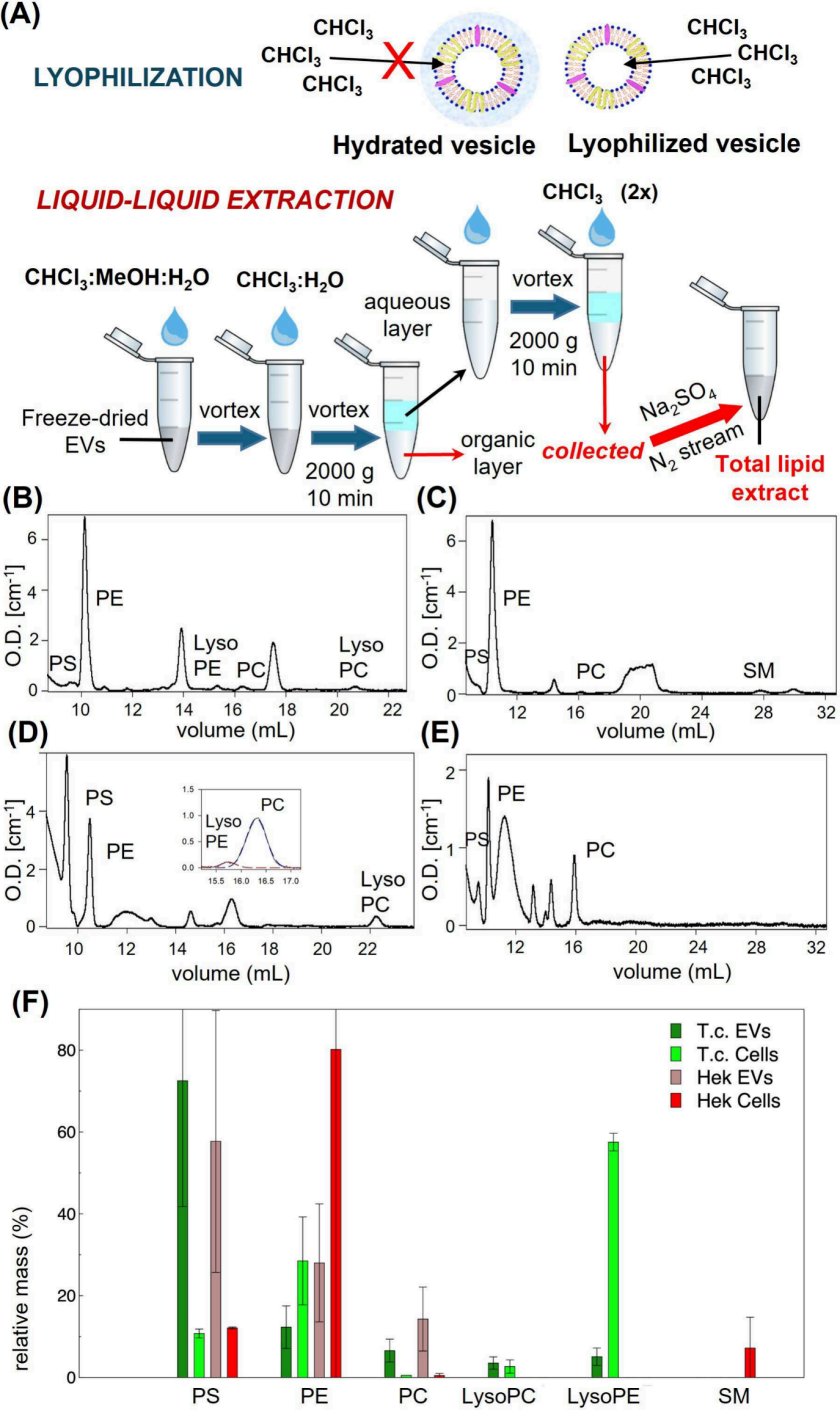
(A) Workflow for lipid extraction combining sample lyophilization and Bligh–Dyer liquid–liquid extraction. (B–E) Representative phospholipid profiles from polar lipid extracts of (B) *T. chuii* cells, (C) HEK293T cells, (D) *T. chuii*-derived EVs, and (E) HEK-derived EVs. (F) Lipid class enrichment in HEK293T and *T. chuii* cells and their corresponding EVs. Data are from three independent biological samples (two for HEK-EVs).

#### HPLC-UV-DAD: Phospholipid Composition of EVs and Parent Cells

The dried lipid extract was resuspended in 100 μL *n*-hexane:2-propanol (3:1, v/v) to be injected in the affinity column. Before injection, each extract was further diluted to achieve optimal peak separation and identification.

Representative chromatograms of each sample are shown in Figure 3(B)–(E). HPLC chromatograms were analyzed to identify the known standards present in the samples. Some peaks were not clearly assigned, or were corresponding to a phospholipid class not included in our pool. Phospholipid content was determined by comparing both the retention times and peak areas of the sample with those of the reference standards. After baseline subtraction, peak fitting was performed to reduce signal fluctuations and to clearly separate closely eluting peaks (as in the inset of Figure 3(D)). Each EVs polar lipid extracts were obtained from three independent EV preparations from source cells, and the data presented corresponds to the average values obtained across these batches. Figure 3(F) shows the results obtained from the analysis of each identified chromatographic peak, comparing the distribution of phospholipids between EVs and their parental cells. Data analysis reveals a striking divergence in lipid composition between EVs and their parental cells, in both mammalian (HEK-293T) and microalgal (*T. chuii*) systems. Notably, PS emerges as a consistently enriched lipid species in EVs derived from both HEK-293T cells and microalgae, in striking contrast to its relatively low abundance in the corresponding parental cells (Figure 3).

More in detail, in nanoalgosomes PS emerged as the predominant phospholipid class, accounting for approximately 68% of the total phospholipid mass on average across three independent batches. In comparison, PS constituted only about 7% of the total molar lipid composition in microalgal cells. A similar enrichment pattern was observed in the mammalian system, where PS represented approximately 56% of the lipid content in HEK-derived EVs, compared to just 17% in the parental HEK-293T cells. Conversely, PE and LysoPE exhibit an opposite distribution pattern compared to PS. In microalgae, LysoPE is a distinctive component of cellular membranes, representing a major lipid class with an average abundance of approximately 67% in parental cells. Although its levels markedly decrease during EV formation, LysoPE remains detectable in nanoalgosomes, where it accounts for around 8% of the total phospholipid content. PE is also present in both *T. chuii* cells and their derived nanoalgosomes, with relative abundances of 21% and 12%, respectively. In HEK-293T cells, PE is the most abundant phospholipid, constituting approximately 81% of the total lipid content. However, its level significantly drops in HEK-derived EVs, where it represents only 29%. Remarkably, LysoPE was not detected in both HEK cells and their corresponding EVs. PC is present in both EVs and parental cells across the two biological systems, and it is enriched in EVs compared to their cellular sources: it accounts for 6% of the total phospholipid content in nanoalgosomes and 14% in HEK-derived EVs, whereas it is present at much lower levels in both microalgal and HEK cells (approximately 0.5%). LysoPC is predominantly found in microalgal cells (3%) and is detectable in nanoalgosomes at trace levels (0.6%) but is absent in the HEK system. Interestingly, sphingomyelin (SM) was detected exclusively in HEK-293T cells and was completely absent in all EV samples, as well as in microalgal cells. This observation aligns with previous reports, and particularly, a recent study also reported the absence of sphingomyelin in T. chuii lipid extracts further supporting our findings.^30^

Altogether, these findings highlight intrinsic differences in the basic lipid composition of the two cell types. Microalgal cells exhibited a higher LysoPE-to-PE ratio, while HEK293T cells showed the opposite, with a strong predominance of PE. This contrast likely reflects distinct metabolic and physiological adaptations: in microalgae, the elevated activity of phospholipase A enzymes, which convert PE into LysoPE, is associated with membrane remodeling and responses to environmental stressors such as light intensity, temperature shifts, or nutrient fluctuations.^31–33^ In contrast, HEK293T cells, grown under controlled and stable culture conditions, maintain a more balanced and less stress-responsive phospholipid profile, resulting in lower levels of LysoPE.^34^

Regarding EVs, the selective enrichment of PS in both EV types likely reflects specific sorting mechanisms involving protein–lipid interactions that favor the incorporation of negatively charged lipids during vesicle formation. This enrichment supports the role of PS as an evolutionary conserved molecular signature of EVs. The preferential accumulation of PS may contribute to defining EV identity, promoting membrane curvature and EV biogenesis, and facilitating cargo recognition and delivery.^6^ PS has been previously implicated in vesicle uptake and signaling in mammalian systems,^7,35^ and its prominent presence in nanoalgosomes extends its potential functional relevance to microalgal EVs.

### ATR-FTIR Spectroscopy Experiments and Analysis

The biochemical profiles of EVs originated from both HEK293T mammalian cells and *T. chuii* microalgal cells were assessed by Fourier Transform Infrared (FTIR) spectroscopy. The experiments were performed on dried sample by using Attenuated Total Reflectance (ATR). While ATR does not allow to measure the absolute amount of the probed component, it is extremely advantageuous since samples do not need to be deuterated and can be used without further preparation.^36^ Figure 4 displays representative spectra of HEK-derived EVs and nanoalgosomes within the 4000–600 cm^–1^ range. The regions typically associated with proteins, lipids, and lipid/carbohydrate/nucleic acids are highlighted in gray.

**Figure 4:**
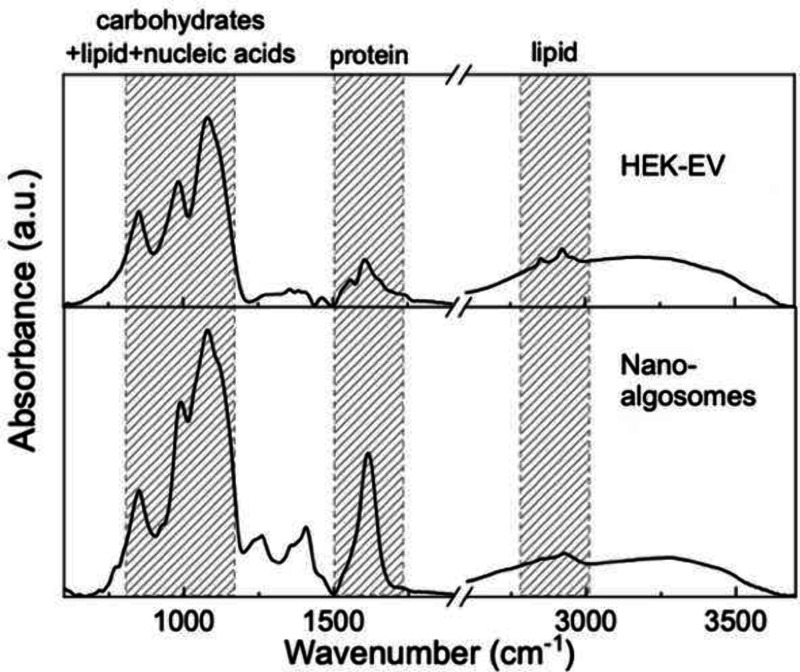
Representative ATR-FTIR spectra of HEK-EVs (top panel) and microalgal-EVs (bottom panel).

Although almost all these spectral features are present in both HEK and microalgal EV samples, the analysis revealed interesting differences in the structural and conformational properties between the two types of extracellular vesicles.

#### Amide I, Amide II, CO Stretching Region (1500–1800 cm^–1^)

The 1500–1700 cm^–1^ region is dominated by the Amide I and Amide II vibrations, the characteristic absorption bands of proteins. The Amide I band mainly arises from C=O stretching, weakly coupled with C–N stretching and N–H bending, and results from the superposition of bands related to specific secondary structures, such as α-helix, β-sheet, β-turn and irregular motifs.^37–39^ The Amide II band, due to C–N stretching and N–H bending, is less sensitive to conformational changes because of overlapping side-chain contributions. In addition to the amide bands, a peak at about 1745 cm^–1^ corresponds to lipid C=O stretching. Given the overlap of these modes, a multicomponent analysis was required to interpret this complex spectral profile. To avoid baseline artifacts, the analysis was extended up to 1780 cm^–1^, thus including the full carbonyl stretch region.

Figures 5(A),(B) show the experimental spectra and corresponding analyses for microalgal and HEK-derived EVs, respectively. In the upper panels, the second derivative spectra are displayed, while the lower panels show the experimental data and Gaussian deconvolution.

**Figure 5:**
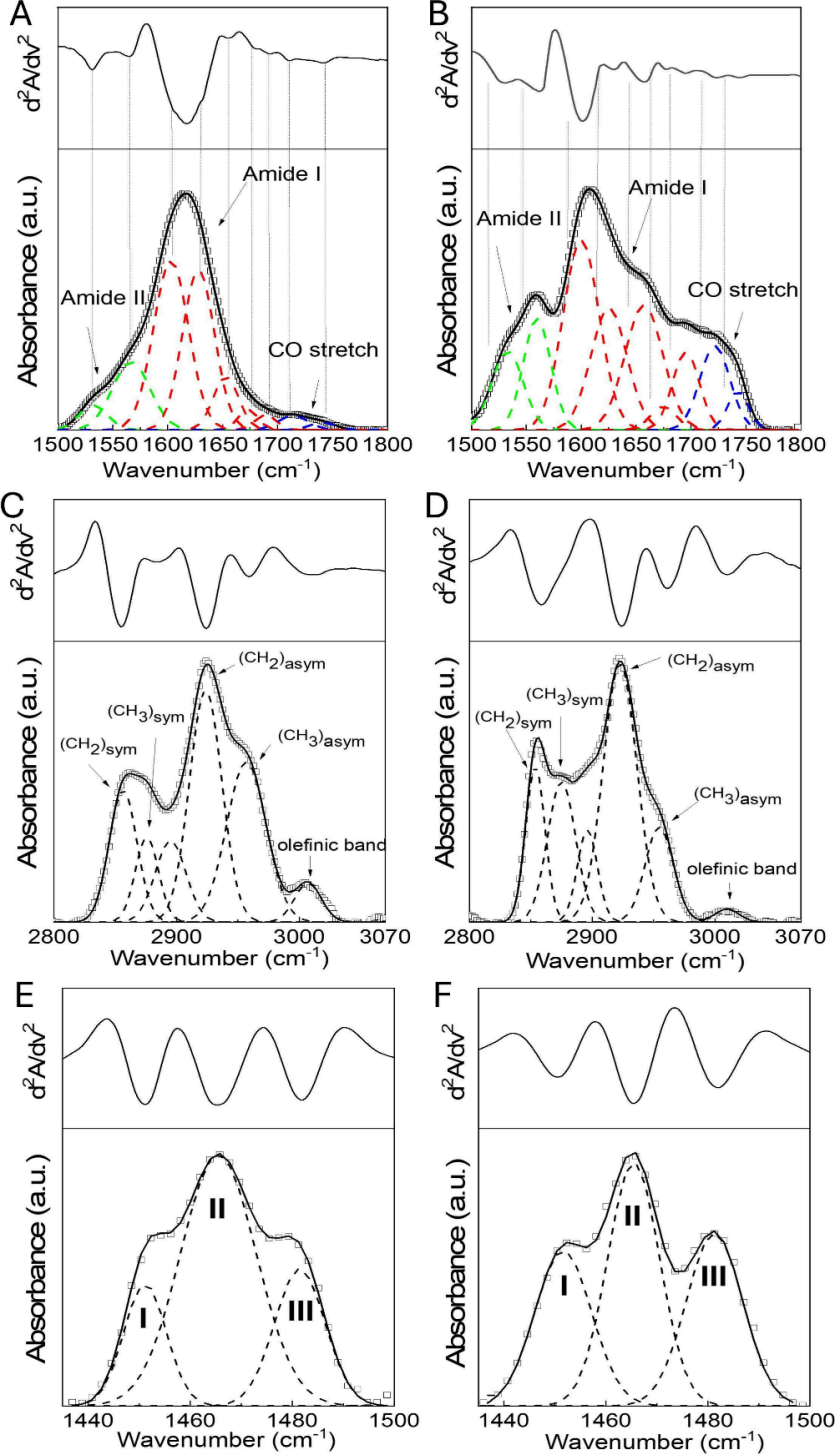
Representative ATR-FTIR spectra and analysis of nanoalgosomes (A, C, E) and HEK-derived EVs (B, D, F) for three different spectral regions: (A, B) amide II, amide I and carbonyl region (1500–1750 cm^–1^); (C, D) CH stretching region (3050–2800 cm^–1^). (E, F) CH_2_ scissoring vibration spectra (1420–1500 cm^–1^). Lower panels: experimental data (squares), best fit (solid lines), and Gaussian components (dashed lines). Upper panels: second derivative spectra.

Second derivative analysis revealed nine Gaussian components in this range. Two bands (1531 and 1565 cm^–1^) are attributed to amino acid side chains within the Amide II region. In the Amide I region (1600–1700 cm^–1^), five distinct bands were observed. The low-frequency component near 1600 cm^–1^ accounts for roughly 35% of the total Amide I area in both EV types and is attributed to “distorted β-type” structures at the ends of β-sheet strands.^40^ Since this band is not directly linked to specific secondary structures, it was excluded from quantitative analysis. The remaining four bands at 1630, 1655, 1675, and 1689 cm^–1^ correspond to parallel β-sheets, α-helix, β-turns, and antiparallel β-sheets, respectively, in good agreement with literature data.^11,13,41–43^

From the integrated areas of the Gaussian components, the relative content of each secondary structure was calculated (Table 2). The total β-sheet content (parallel + antiparallel) is similar in both EV types, whereas α-helix and β-turn fractions differ markedly. HEK-EVs show a higher proportion of α-helices and a lower content of β-turns. Since α-helices and β-sheets are typical of ordered protein structures, while β-turns indicate conformational flexibility,^44^ HEK-EVs can be regarded as possessing a higher degree of protein structural order.

**Table 2:** Results of the Amide I Peak Deconvolution: Peak Positions, Secondary-Structure Assignment and Relative Areas for HEK-EVs and Microalgal-EVs (mAlg-EVs)

Peak (cm^–1^)	Secondary structure	HEK-EVs area (%)	mAlg-EVs area (%)
1630	parallel β-sheets	9.8 ± 0.3	11.58 ± 0.07
1656	α-helix	10.5 ± 0.5	2.16 ± 0.02
1675	β-turns	15.5 ± 0.3	17.07 ± 0.14
1689	antiparallel β-sheets	16.0 ± 0.3	1.90 ± 0.02

Regarding the carbonyl stretching band, two components were identified around 1726 and 1744 cm^–1^ in all samples. Such a doublet has been previously reported and used to probe phase behavior and headgroup interactions in lipids.^11,45^ In our samples, however, the C=O band intensity is significantly reduced compared with pure lipids and is weaker in microalgal EVs than in HEK-EVs. This attenuation likely reflects factors intrinsic to extracellular vesicles, such as intermolecular interactions, molecular orientation, and packing constraints, which limit carbonyl mobility and reduce vibrational intensity. A comparable reduction of the C=O stretching band in extracellular vesicles was reported by Mihály et al. (2017),^11^ who attributed it to lipid composition differences between large EVs (pelleted at 20,000 × g and including microvesicles) and small EVs (pelleted at 110,000 × g and including exosomes), the latter showing almost complete suppression of the band, and a higher β-turn content. Taken together, our observations may indicate a higher proportion of small-EV-like elements within the microalgal EV samples.

#### C–H Stretching Region (2800–3050 cm^–1^)

At the highest frequencies, all spectra exhibit a broad band between 3800 and 3000 cm^–1^, attributed to overlapping O–H and N–H stretching vibrations mainly from carbohydrates and proteins (Figure 4). At lower frequencies, the 2800–3050 cm^–1^ region is dominated by C–H stretching vibrations typical of lipids. Figures 5(C),(D) show representative spectra for both HEK- and microalgal-derived EVs, with second derivatives (upper panels) and Gaussian deconvolutions (lower panels).

This spectral window includes CH_2_ symmetric stretching (∼2850 cm^–1^, (*CH*_2_)_*sym*_), CH_2_ asymmetric stretching (∼2920–2924 cm^–1^, (*CH*_2_)_*asym*_), CH_3_ symmetric stretching (∼2870 cm^–1^, (*CH*_3_)_*sym*_), and CH_3_ asymmetric stretching (∼2954 cm^–1^, (*CH*_3_)_*asym*_).^46–49^ A weak olefinic band at ∼3005 cm^–1^, arising from HC=CH vibrations of unsaturated fatty acids (UFA), is observed in both vesicle types.^11,13,45,50^ Conversely, the symmetric and asymmetric CH_2_ stretching modes are typical of saturated fatty acids (SFA), hereafter labeled as SFA1 and SFA2, respectively.^13,43,51^

Overall, the analysis of this region clearly differentiates the spectroscopic contributions of saturated and unsaturated fatty acids in both EV species, providing a reliable molecular fingerprint of their lipid composition.

#### CH_2_ Scissoring Region (1400–1500 cm^–1^)

The CH_2_ scissoring vibration band provides valuable information on interchain interactions and the lateral packing of lipid molecules. Depending on lipid composition, lateral organization may adopt orthorhombic (OR), hexagonal (HEX), or liquid-like (LIQ) arrangements.^14,46,52–54^

In the orthorhombic phase, aliphatic chains are fully extended (all-trans conformation) and tightly packed in a rectangular lattice. The hexagonal phase exhibits looser packing with more gauche conformations, whereas the liquid-disordered phase shows largely lost lateral order. These phases are readily distinguished by IR spectroscopy: hexagonal packing produces a single band near 1467 cm^–1^, while orthorhombic packing causes short-range coupling between chains, splitting the scissoring band into two additional peaks at slightly higher and lower wavenumbers

Based on these considerations, we analyzed the CH_2_ scissoring region (1430–1500 cm^–1^) using second-derivative and Gaussian deconvolution, as shown in Figures 5(E),(F) for representative HEK- and microalgal-derived EVs. The spectra clearly display three components, confirming the coexistence of mixed lipid phases in all samples. The peaks at ∼1453 and ∼1481 cm^–1^ (I and III in Figures 5(E),(F)) correspond to the orthorhombic phase, whereas the one at ∼1466 cm^–1^ (II) represents the hexagonal phase The percentage area of each component was obtained as the ratio between its integrated area and the total CH_2_ scissoring band area, providing a quantitative estimate of the relative abundance of the different lipid packing phases.

#### Spectroscopic Indicators

We may consider the total area of the spectra between 2800 and 3000 cm^–1^ as a measure of the total lipid content. C–H stretching modes are widely used to assess lipid order, as their frequency shifts correlate with trans–gauche isomerization and acyl chain mobility.^46,50,54,55^ Additionally, the integrated areas of these bands provide information on acyl chain length, degree of saturation, and overall membrane organization. Starting from these considerations, several spectroscopic indicators were evaluated to compare the structural properties of HEK- and microalgal-derived EVs.

##### (1) Protein/Lipid Ratio

Among all parameters, the “spectroscopic” protein-to-lipid ratio (P/L) represents a key marker of EV composition and functionality. Originally introduced by Navarro^56^ and later refined by Mihály and Stepien,^11,13^ it is obtained by dividing the integrated area of the amide I band (protein contribution) by that of the lipid region, excluding nonvesicular components such as aggregated proteins or free amino acids, if any (accordingly, bands below 1615 cm^–1^ were excluded):

4 $$P/L=\frac{A_{\mathrm{amide I}}}{A_{\mathrm{CH stretching}}}$$

where *A*_amide I_ is the area of the bands peaked between 1615 and 1700 cm^–1^ and *A*_CH stretching_ is the area of the bands peaked between 2800 and 3000 cm^–1^

The calculated P/L ratios were ∼0.63 for HEK-EVs and ∼0.95 for microalgal-EVs (Figure 6). Although these are not absolute values, two conclusions can be drawn: (i) in both vesicle types, the protein fraction is lower than the lipidic one, consistent with a bilayer structure; (ii) HEK-EVs show a lower P/L ratio, typical of large vesicles, whereas the higher ratio of microalgal-EVs suggests a population enriched in small vesicles with higher protein cargo, as confirmed by DLS measurements (Figure 1).

**Figure 6:**
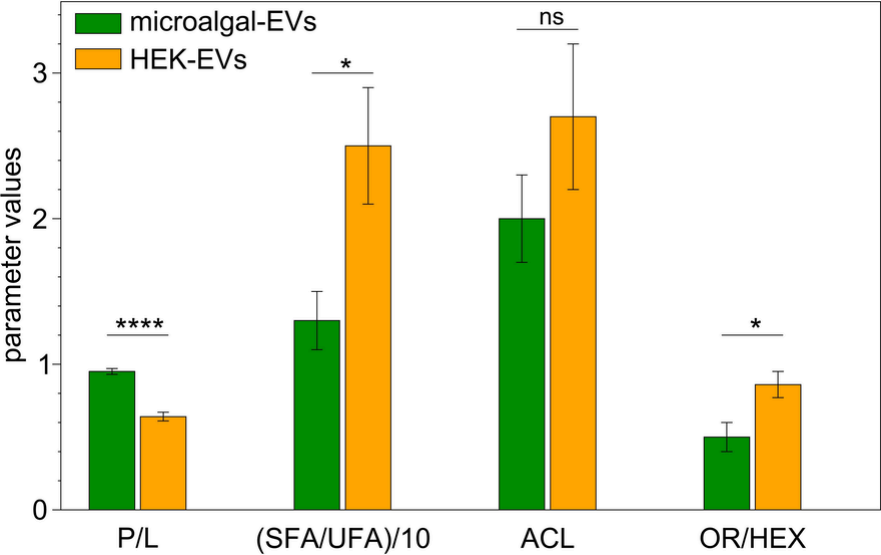
Spectroscopic indicators for HEK-EVs and microalgal-EV: protein/lipid ratio (P/L), saturated fatty acid to unsaturated fatty acid ratio (SFA/UFA), acyl chain length (ACL), orthorhombic phase to hexagonal phase ratio (OR/HEX). The statistical significance of differences between the two EV subtypes was assessed using two-tailed unpaired t-test and evaluating the p values: ****p < 0.0001; *p < 0.05; ns, not significant.

Orthogonal and independent biochemical and chromatographic measurements (namely BCA assays, lipid extract mass, phospholipid content) support the interpretation of this FTIR-derived indicator (Figure S1, Supporting Information) and encourage its use as a semiquantitative descriptor for comparative EV characterization.

##### (2) Saturated/Unsaturated ratio and (3) Acyl Chain Length

Further insight into EV membrane organization was obtained by analyzing the CH stretching region to derive two complementary indicators: the saturated-to-unsaturated fatty acid ratio (SFA/UFA) and the acyl chain length (ACL).

The SFA/UFA ratio was computed as the total area of the saturated components (SFA1 + SFA2) divided by that of the unsaturated one (UFA):

5 $$\mathrm{SFA}/\mathrm{UFA}=\frac{A_{\mathrm{SFA}1}+A_{\mathrm{SFA}2}}{A_{\mathrm{UFA}}}$$

where *A*_SFA1_ and *A*_SFA2_ are the area of the bands peaked at 2850 cm^–1^ (*CH*_2_ symmetric stretching) and at 2922 cm^–1^ (*CH*_2_ asymmetric stretching, respectively, and *A*_UFA_ is the area of the bands peaked at 3005 cm^–1^ (olefinic band).

The ACL parameter was obtained as the ratio of the area under the (*CH*_2_)_asym_ band, peaked at 2922 cm^–1^, (SFA2) to that under the (*CH*_3_)_asym_ band, peaked at 2954 cm^–1^,:^13,45,55^

6 $$\mathrm{ACL}=\frac{A_{{\left({\mathrm{CH}}_{2}\right)}_{\mathrm{asymmetric}}}}{A_{{\left({\mathrm{CH}}_{3}\right)}_{\mathrm{asymmetric}}}}$$

We do not include in this calculation the areas of the (*CH*_2_)_*sym*_ and the (*CH*_3_)_*sym*_ bands as in other studies,^57^ since they are more overlapped and less intense. The evaluated parameters are reported for each sample in Figure 6.

Both parameters were consistently lower in microalgal-EVs than in HEK-EVs, indicating the presence of shorter and more unsaturated acyl chains in the former. These structural features are functionally relevant, as acyl chain length and saturation collectively govern membrane packing and mechanical behavior.^58^ Longer, more saturated chains favor compact and rigid bilayers, whereas unsaturation and shorter chains introduce conformational disorder and enhance membrane fluidity. The lower ACL observed in microalgal-EVs therefore reflects a more flexible and dynamic membrane structure compared with HEK-EVs.

Notably, the ACL is not merely a geometric descriptor: it represents an effective, spectroscopically derived average of the methylene-to-methyl ratio in the lipid matrix, providing a direct indicator of lipid order at the molecular scale and allowing cross-validation with chromatographic results when available (e.g., HPLC-DAD data on fatty acid composition). As discussed elsewhere,^11,13,45,55^ a lower ACL corresponds to a higher proportion of short-chain or unsaturated lipids, which typically reduce van der Waals coupling and increase membrane permeability and fluidity. In this respect, the ACL parameter offers a quantitative bridge between the spectroscopic and biochemical characterization of extracellular vesicle membranes.

Further evidence of a more disordered membrane structure in microalgal-EVs is provided by the position of the (*CH*_2_)_*asym*_ band (SFA2), which appears at slightly higher wavenumbers compared to HEK-EVs. The frequencies of both symmetric and asymmetric CH_2_ stretching modes are highly sensitive to the conformational state of lipid acyl chains.^50,51,59^ A shift toward higher frequencies indicates a transition from an ordered, all-trans configuration of hydrocarbon chains to a more disordered, liquid–crystalline state enriched in gauche conformers. This spectral behavior is fully consistent with the lower ACL and SFA/UFA ratios observed in nanoalgosomes, confirming their increased acyl chain disorder and higher membrane fluidity.

A final consideration concerns the link between lipid disorder and protein–lipid interactions. Previous studies have shown that membranes with a higher protein content tend to incorporate a greater proportion of shorter and more disordered acyl chains, as this composition optimizes local packing and enhances protein–lipid coupling.^6,14,60^ Notably, other studies also reported lower heterogeneity in the lipid bilayer structure of small EVs, with respect to large EVs, consistent with their more optimized local packing.^61^

Our findings are consistent with this view: samples exhibiting lower ACL values — namely the nanoalgosomes — also display higher protein-to-lipid ratios, suggesting that enhanced membrane fluidity facilitates protein embedding and stabilization within the bilayer.

Despite the apparent trend discussed above, it should be noted that the ACL difference between the two EV types is not statistically significant (Figure 6), indicating that in this case the ACL indicator primarily captures a trend rather than a statistically resolved distinction in the present data sets.

##### (4) Orthorhombic/Hexagonal Phase

Additional information on lipid packing can be obtained from the CH_2_ scissoring bands, specifically through the ratio between the orthorhombic (OR) and hexagonal (HEX) components (OR/HEX). These components were quantified as (HEX) the area of peak II (*A*_1466*cm* ^–1^_) and (OR) the sum of the areas of peaks I (*A*_1453*cm* ^–1^_) and III (*A*_1481*cm* ^–1^_), respectively:

7 $$\mathrm{OR}/\mathrm{HEX}=\frac{A_{1453{\mathrm{cm}}^{-1}}+A_{1481{\mathrm{cm}}^{-1}}}{A_{1466{\mathrm{cm}}^{-1}}}$$

As shown in Figures 5(E), (F) and in the histograms of Figure 6, nanoalgosomes are mainly organized in a loosely packed, disordered hexagonal structure, with a smaller fraction of orthorhombic phase. In contrast, HEK-derived EVs display both phases in nearly equal proportions.

These findings reinforce the evidence obtained from the SFA/UFA and ACL parameters, confirming that the lipid matrix of microalgal-EVs is characterized by higher conformational disorder and greater lateral mobility. Such packing differences reflect the molecular composition of the acyl chains: shorter and more unsaturated hydrocarbons weaken van der Waals interactions, increasing interchain spacing and favoring a transition from orthorhombic to hexagonal arrangements.^46,60^

Taken together, these results indicate that nanoalgosomes exhibit a more fluid and dynamic lipid phase organization than HEK-derived EVs, a property that may influence their stability, membrane permeability, and capacity for molecular exchange.

#### Carbohydrates, Lipids, and Nucleic Acids Region (1000–1200 cm^–1^)

The spectral region below 1400 cm^–1^ encompasses several overlapping vibrational modes. Bands between 1200 and 1400 cm^–1^ arise from a combination of N–H bending and C–N stretching, along with deformation vibrations of C–H and N–H groups. At lower wavenumbers, the 1200–900 cm^–1^ range is mainly associated with vibrations of carbohydrates, lipids, and nucleic acids.^11,41,43,48,69^

This spectral window is the most complex among those investigated, as it results from a strong overlap of multiple components arising from coexisting carbohydrates, lipids, and nucleic acids. To achieve a more reliable band discrimination, the second-derivative analysis was complemented by the fourth derivative, which enabled the resolution of weak or hidden components that were later confirmed by Gaussian deconvolution. Figure 7 shows, from top to bottom, the fourth-derivative, second-derivative, and FTIR-ATR absorption spectra along with the corresponding deconvolution for representative EV samples. The close match between derivative-identified and deconvoluted bands supports the robustness of the spectral analysis. Despite the intrinsic complexity of this region, a consistent spectral pattern was observed among vesicles of the same species.

**Figure 7:**
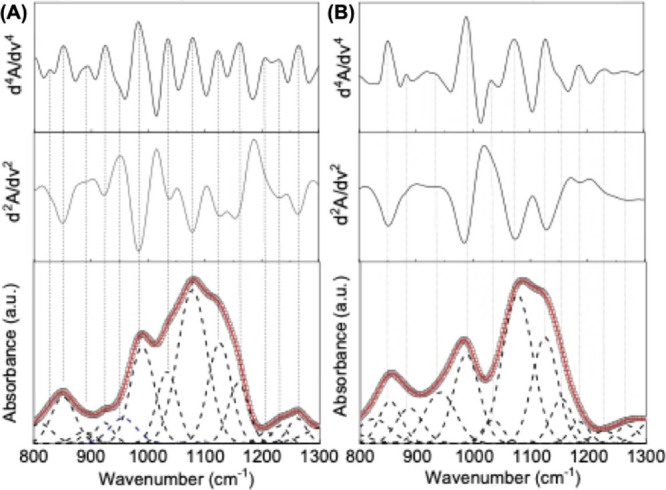
Representative ATR-FTIR spectra and analysis of nanoalgosomes (left panels) and HEK-derived EVs (right panels) in the region 800–1300 cm^–1^. Lower panel: experimental data (squares), best fit (solid lines), and Gaussian components (dashed lines). Middle panels: corresponding second derivative spectra. Upper panels: corresponding fourth derivative spectra.

Table 3 summarizes the mean peak positions and percentage areas of the main components for both EV types, together with tentative assignments based on literature data. Several components are common to both species and contribute similarly to the overall spectral profile, yet distinct differences highlight their compositional diversity. In particular, characteristic DNA-related bands (886 and 1183 cm^–1^) were detected in HEK-EVs but were absent in microalgal EVs. This difference agrees with their distinct biogenesis pathways.Although mammalian EVs have been reported to contain DNA cargo, including genomic and mitochondrial DNA fragments,^2^ plant- and microalgae-derived EVs are predominantly described as carriers of proteins, lipids, metabolites and small RNAs, with no consistent evidence for substantial DNA content.^20,72^ This difference is consistent with the absence of detectable DNA-associated bands in microalgal EV spectra.In contrast, absorption bands ascribed to RNA vibrations (at approximately 1124, 1076, and 988 cm^–1^) were evident in both EV types and accounted for a substantial portion of the total spectral profile. This strong RNA contribution supports the view that RNA is a conserved and abundant molecular component in extracellular vesicles, independent of cellular origin.^42,48^

**Table 3:** Peak Frequency, Relative Area and Tentative Assignment of Major Spectral Components from Gaussian Deconvolution for HEK–EVs and Microalgal EVs

HEK-EV		microalgal-EV			
Wavelength (cm^–1^)	Area (%)	Wavelength (cm^–1^)	Area (%)	Proposed assignment	refs.
850	5.4	850	6.9	C–O stretch, C–O–C bending	^62^,^63^
				(carbohydrates/sugar-phosphate backbone)	
886	5.3	–	–	Deoxyribose ring vibration	^43^,^64^
–	–	923	2.2	β-glucosidic linkage (carbohydrates)	^63^,^65^
940	9.0	–	–	C–O bonds (carbohydrates);β-DNA helical form	^63^,^64^,^66^
–	–	955	4.3	Skeletal mode of glycosidic linkage (carbohydrates)	^63^,^66^
986	13.0	989	14.5	Ribose-phosphate main chain (RNA);COH, COC, CH_2_ stretching	^15^,^63^,^66^
 ^42,67–69^					
1031	2.6	1033	9.3	C–O, C–C stretching;	
				COH vibration (carbohydrates)	^63^,^65^,^70^
1075	25.7	1077	28.4	Symmetric phosphodiester stretching	^42^,^43^,^67^
				(phospholipid, RNA/DNA)	^15^,^69^
					^51,64^
1123	15.4	1125	14.6	PO_2_ stretching of phosphodiester backbone;	^63^,^66^
				C–O stretching of ribose/deoxyribose (nucleic acids);	^67–69^
				C–O bending (carbohydrates)	
1152	5.5	1157	7.6	CO, CC stretching;	^15^,^43^,^63^
				COH deformations (carbohydrates)	^65–68^
1183	2.3	–	–	Stretching of the sugar–phosphate backbone	^70^,^71^
				(A-type deoxyribose)	
1230	1.6	1230	1.7	Asymmetric phosphodiester stretching	^43^,^64^,^68^
				(phospholipid, DNA)	^15^,^69^

Most bands in this region arise from overlapping contributions of multiple molecular groups, as clearly shown by the tentative assignments reported in the Table 3. This makes the bands at 850 and 1030 cm^–1^ particularly interesting, as they appear to be specifically associated with the presence of sugars. More specifically, they are assigned to C–O stretching and C–O–H bending vibrations presumably associated with glycolipids, glycoproteins, or phosphate-sugar structures, or also with microalgal polysaccharides for nanoalgosomes. Interestingly, among all the bands analyzed, the one at 1030 cm^–1^ exhibited the most pronounced variation in percentage area (approximately 7%) between the two EV species (thus potentially representing a differential marker). Although the higher sugar content in microalgal EVs is evident, a more accurate assessment was obtained by normalizing the area of this band to that of the PO_2_ symmetric stretching (∼1076 cm^–1^). The higher ratio observed in algal EVs compared to HEK EVs confirms a greater polysaccharide content in the vesicles derived from microalgae.

### EV Fatty Acid Composition

Having established the conserved PS EV signature (HPLC-DAD) and specific differences in acyl chain saturation (FTIR) across human and microalgal EVs, we applied Gas Chromatography–Mass Spectrometry (GC-MS) to complete the biochemical fingerprint. The GC-MS step provides the necessary molecular breakdown to validate the saturation and length indicators suggested by FTIR and definitively quantify the entire fatty acid profile of the nanoalgosomes.

Fatty acids were extracted from phospholipids, methylated, and subsequently identified by GC-MS (Quarta et al., 2024). The analysis of Fatty Acid Methil Esthers (FAMEs) obtained from lipid extracts revealed a heterogeneous profile in terms of saturation and chain length (Table 4).

**Table 4:** Fatty Acid Methyl Esters Identified by GC–MS with Retention Times, Molecular Formulas, Calculated and Experimental Molecular Weights, and Relative Abundances

Fatty acid methyl esters	Ret. time (min)	molecular formula	short-hand notation	MW (Da) calc.^a^	MW (Da) exp.^b^	microalgal EVs Area (%)	HEK EVs Area (%)
Pentadecanoic Acid	12.79	C_16_H_32_O_2_	15:0	256.2402	256.34	2.1 ± 0.7	-
7-Hexadecenoic acid	13.65	C_17_H_32_O_2_	16:1	268.2402	268.45	1.3 ± 0.5	15.5 ± 6.2
Palmitoleic Acid^c^	13.71	C_17_H_32_O_2_	16:1	268.2402	268.43	1.8 ± 0.9	1.9 ± 1.2
Palmitic Acid^c^	13.96	C_17_H_34_O_2_	16:0	270.2558	270.29	8.7 ± 5.1	43.0 ± 6.4
Heptadecanoic acid	14.65	C_18_H_36_O_2_	17:0	284.2715	284.38	15.0 ± 7.2	5.4 ± 0.6
9,12-Linoleic Acid^c^	15.65	C_19_H_34_O_2_	18:2	294.2558	294.47	9.1 ± 6.8	5.3 ± 0.4
Linoleic acid isomer	15.69	C_19_H_34_O_2_	18:2	294.2558	294.42	4.1 ± 0.6	2.2 ± 1.4
Oleic Acid^c^	15.79	C_19_H_36_O_2_	18:1	296.2715	296.49	45 ± 18	8.8 ± 1.6
Oleic Acid isomer	15.84	C_19_H_36_O_2_	18:1	296.2715	296.49	-	3.4 ± 0.7
Stearic Acid^c^	15.99	C_19_H_38_O_2_	18:0	298.2871	298.51	3.0 ± 0.8	11.5 ± 3.0
Linolelaidic acid	16.15	C_19_H_34_O_2_	18:2	294.2558	294.43	0.8 ± 0.8	-
Nonadecanoic acid	16.33	C_20_H_40_O_2_	19:0	312.3028	312.52	0.1 ± 0.1	3.1 ± 0.6
Eicosenoic acid	17.59	C_21_H_40_O_2_	20:1	324.3028	324.56	0.2 ± 0.2	-
Eicosanoic acid	17.81	C_21_H_42_O_2_	20:0	326.3184	326.56	1.8 ± 0.3	-
DHA^c^	18.99	C_23_H_34_O_2_	22:6	342.2558	342.51	6.7 ± 2.3	-

a Calculated from https://www.sisweb.com/referenc/tools/exactmass.htm.

b Experimentally determined as M^+^.

c Peak assigned by using the corresponding analytical standard as in the Materials and Methods section.

Among the identified components,palmitic acid (C16:0) was the most abundant in HEK-derived EVs, followed by 7-Hexadecenoic acid (C16:1) and the saturated stearic acid (C18:0). In microalgal-EVs, the unsaturated components are more represented. In particular,oleic acid (C18:1) was the most abundant component accounting for approximately 45% of the total fatty acid content. Heptadecanoic acid (C17:0) and linoleic acid (C18:2,9,12) were also present in significant amounts. Saturated fatty acids such as palmitic acid (C16:0) and stearic acid (C18:0) were detected at 8.1% and 2.8%, respectively. Minor components included omega-6 and omega-3 fatty acids such as methyl linoleate isomer (3.8%), methyl linolelaidate (2.4%), and docosahexaenoic acid (DHA, C22:6), which was present at 6.2%. Trace levels of odd-chain and long-chain saturated and monounsaturated fatty acids such as nonadecanoic acid (C19:0), eicosenoic acid (C20:1), and eicosanoic acid (C20:0) were also identified.

In contrast, the microalgal FAME profile as shown in our previous work (Adamo et al., 2021) exhibited greater diversity in unsaturated fatty acids, especially long-chain PUFAs. Fatty acids typical of microalgal membrane lipids-such as C16:4, C18:3, C18:4 were identified, as well as the presence of eicosapentaenoic acid (EPA, C20:5), known for its beneficial nutraceutical and pharmacological properties. These results indicate that microalgae-derived lipids are enriched in monounsaturated and polyunsaturated fatty acids, in tune with FTIR results, particularly oleic and linoleic acids, which may contribute to their biophysical properties and potential biological activity.

Using these results, we calculated the ratio between saturated and unsaturated fatty acids (SFA/UFA), as well as an approximate geometrical estimate of the acyl chain length. These quantities correspond to the FTIR-derived SFA/UFA and ACL indicators introduced in the previous section. Although obtained using different analytical approaches, they show consistent trends, thus providing orthogonal support for the interpretation of the semiquantitative spectroscopic parameters (Figure S2, Supporting Information).

## Conclusions

In this study we established a multitechnique strategy to investigate the lipid composition and structural organization of extracellular vesicles from mammalian (HEK293T) and microalgal (*T. chuii*) cells. The integration of HPLC-DAD, ATR-FTIR and GC–MS allowed us to highlight both conserved and species-specific lipid features, while demonstrating the value of combining chromatographic, mass spectrometric, and spectroscopic approaches for EV characterization.

Our data revealed phosphatidylserine as a conserved and selectively enriched lipid in vesicles across these phylogenetically distant species, reinforcing the concept of regulated lipid sorting during the evolutionary conserved process of vesicle biogenesis. The fatty acid profile of nanoalgosomes, enriched in mono- and polyunsaturated chains, was consistent with a bilayer structure that is more fluid and conformationally dynamic compared to HEK-derived EVs.

A distinctive contribution of this work lies in the exploitation of ATR-FTIR spectroscopy beyond general fingerprinting, using it to extract spectroscopic indicators directly related to vesicle structure and composition. Parameters such as the protein-to-lipid ratio, the saturated-to-unsaturated lipid balance, the average acyl chain length, and the orthorhombic-to-hexagonal packing ratio proved effective in discriminating between mammalian and algal EVs. Independent biochemical and chromatographic measurements (Figures S1 and S2, Supporting Information) further support the interpretation of these FTIR-derived indicators. Together, these descriptors indicate that nanoalgosomes display a higher relative protein content, shorter and more unsaturated acyl chains, and a looser membrane packing, with respect to human cell-derived EVs. Such features not only distinguish their biophysical properties but may also underlie specific biological functions, such as enhanced flexibility and interaction capacity.

Recent advances in mass spectrometry imaging approaches, including time-of-flight secondary ion mass spectrometry (ToF-SIMS), are further expanding the lipidomic toolbox by enabling spatially resolved analyses of membrane structures and nanoscale lipid organization.^73^ In this context, the present workflow should be viewed as complementary, providing rapid bulk structural descriptors that can be integrated with emerging high-resolution MS-based methodologies.Our framework therefore complements high-resolution lipidomics, and supports our working hypothesisthat rapid, nondestructive spectroscopic readouts—integrated by HPLC-DAD and GC–MS—can translate complex biochemical profiles into reproducible, functional descriptors. They offer a rapid, nondestructive layer of information to (i) flag sample-to-sample drifts in manufacturing, (ii) discriminate EV sources, even across distant phylogenetic origins, by their membrane organization, order and packing, and (iii) formulate testable hypotheses on uptake, stability, and cargo compatibility grounded in physical membrane parameters. The identification of nanoalgosome-specific lipids of high biological value, together with the spectroscopic descriptors introduced here, strengthens their emerging role as sustainable, tunable nanobiotechnological platforms.

More broadly, the integrated workflow proposed here outlines a flexible and practical route for comparative EV studies and for establishing quality control parameters and indicators. In this perspective, this methodology can support standardization and comparability across different laboratories and production scales, and guide process optimization in EV biomanufacturing.

## Supplementary Material



## Notes

Additional analyses performed to compare ATR-FTIR-derived indicators with independent measurements, including protein-to-lipid ratio estimates from BCA assays and lipid mass measurements (Figure S1); lipid saturation and acyl chain length parameters derived from GC–MS analysis (Figure S2) (PDF)
