# Supplementary material for: Expanding the Analytical Toolbox for Extracellular Vesicle Biochemical Profiling: A Multiplatform Spectroscopic and Chromatographic Strategy
Source: Anal Chem. 2026 Apr 24;98(18):13434–49. doi: 10.1021/acs.analchem.5c07632 (PMC13177279; doi:10.1021/acs.analchem.5c07632)
Supplement: Supplementary file 1 [file ac5c07632_si_001.pdf]

Supporting Information:

Expanding the Analytical Toolbox for Extracellular Vesicle Biochemical Profiling: A Multi-Platform Spectroscopic and Chromatographic Strategy

Caterina Branca,[†,‖] Angela Paterna,[‡,‖] Estella Rao,[‡] Samuele Raccosta,[‡] Mohamed Zekri,[†] Sabrina Picciotto,[¶,‡] Paola Gargano,[¶] Giorgia Adamo,[¶] Luana Pulvirenti,[§] Laura Siracusa,[§] Antonella Bongiovanni,[¶] and Mauro Manno[*,‡]

†*Department of Mathematical and Computational Sciences, Physical Science and Earth Science, 98166 Messina, Italy*

‡*National Research Council of Italy, Institute of Biophysics, 90146 Palermo, Italy;*

¶*National Research Council of Italy, Institute for Biomedical Research and Innovation, 90146 Palermo, Italy;*

§*National Research Council of Italy, Institute of Biomolecular Chemistry, 95126 Catania, Italy*

‖*These authors contributed equally to this work*

E-mail: mauro.manno@cnr.it

Table of Contents

Supporting Figures/Sections	Page numbers
Figure S1: Evaluation of protein to lipid ratio.	S-2
Figure S2: Evaluation of parameter related to lipid saturation and length	S-3

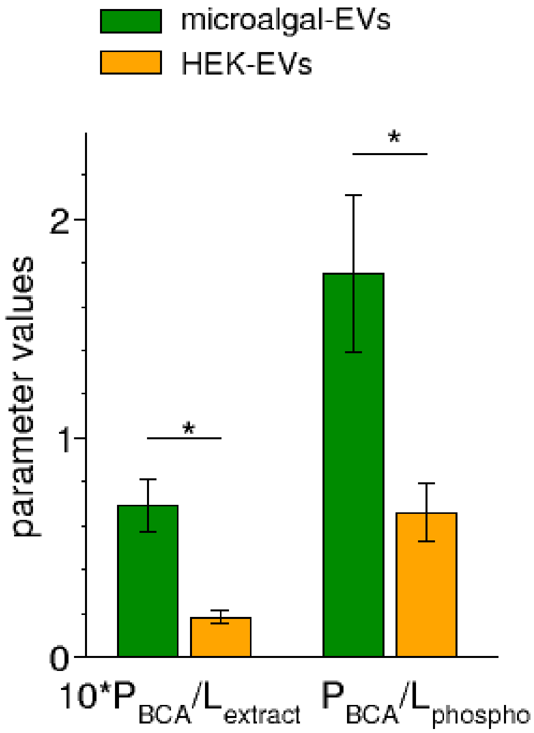

Figure S1: Evaluation of protein to lipid ratio.
P_{BCA} is the total protein amount of EVs, measured in 1 mL solution, determined by BCA assay (in Materials and Methods). The lipid amount was estimated using two parameters. $L_{extract}$ is the mass of the hydrophobic extract obtained by liquid-liquid partitioning (Methods section and Figure 3A). $L_{phospho}$ is the total phospholipid amount quantified by the HPLC-DAD (Results section).
In the batches analysed in this study the following values were obtained: microalgal-EVs: P_{BCA} = 130, 80, 62 μg, $L_{extract}$ = 1.5, 1.2, 1.1 mg, $L_{phospho}$ = 82.8, 55.2, 27.6 μg; HEK-EVs: P_{BCA} = 14.0, 26.3μg, $L_{extract}$ = 0.8, 1.5 mg; $L_{phospho}$ = 22.9, 36.8 μg.
These two lipid estimates do not correspond exactly to total lipid mass. $L_{extract}$ may overestimate lipid mass due to residual solvent in the dried extract, whereas, $L_{phospho}$ represents only a fraction of the total lipid mass, since HPLC-DAD quantifies phospholipid species only. Despite these limitations, both parameters show trends consistent with the FTIR-derived protein-to-lipid ratio P/L (Figure 6).
The statistical significance of differences between the two EV subtypes was assessed using two-tailed unpaired t-Test and evaluating the p values: (* $p < 0.05$).

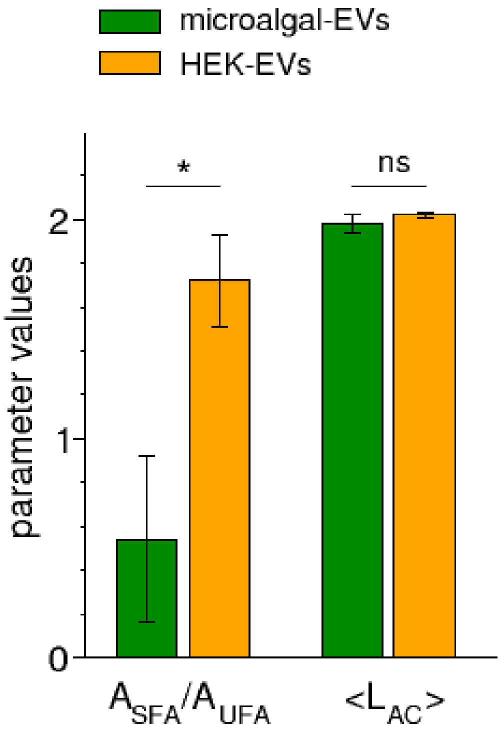

Figure S2: Evaluation of parameter related to lipid saturation and length.
The ratio A_{SFA}/A_{UFA} is calculated from GC-MS measurement as the ratio between the summed integrated peak areas of identified Saturated Fatty Acids A_{SFA} and Unsaturated Fatty Acids A_{UFA}, respectively. The absolute numerical values differ from those obtained by ATR-FTIR, because the two approaches quantify the parameter through different analytical principles. Nevertheless, the trends observed in the GC–MS-derived ratios are consistent with the FTIR-derived indicators – specifically the enrichment of saturated fatty acids relative to unsaturated fatty acids in mammalian EVs compared to microalgal EVs – and therefore provide orthogonal support for the interpretation of the spectroscopic parameter.
The average length of acyl chains $< L_{AC} >$ was estimated by assuming a given length for each fatty acid chain and weighting it with the percentage area measured by GC-MS. The length l_n of a n-carbon saturated fatty acid can be calculated straightforwardly by geometrical construction, by considering the length of a $C-C$ bond ($d_{C-C} = 0.154nm$), the tetrahedral bond angle ($\theta = 109.5°$: $l_n = d_{C-C} + (n-1)d_{C-C}sin(\theta/2)$. The presence of a double bond $C = C$ introduces a local shortening of the effective chain length, which is not immediately predictable. In this worh, we approximate this effect by assuming an *ad hoc* reduction of 0.25 nm for each double bond. The difference between the two EV subtypes is not relevant and not statistically significant, in agreement with the trend obtained from the FTIR-derived parameter (ACL), supporting the interpretation of the spectroscopic ACL parameter as a structural descriptor of lipid chain organization.
The statistical significance of differences between the two EV subtypes was assessed using two-tailed unpaired t-Test and evaluating the p values: (* $p < 0.05$, ns: not significant).
